# Effects of mental fatigue on perception of effort and performance in national level swimmers

**DOI:** 10.3389/fpsyg.2025.1520156

**Published:** 2025-04-09

**Authors:** Dalton de Lima-Junior, Giuseppe Caporaso, Matteo Cortesi, Leonardo de Sousa Fortes, Samuele Maria Marcora

**Affiliations:** ^1^Department for Life Quality Studies, University of Bologna, Bologna, Italy; ^2^Liceo Alessandro Volta, Como, Italy; ^3^Department of Physical Education, Federal University of Paraiba, João Pessoa, Brazil

**Keywords:** cognitive effort, self-regulation, rating of perceived exertion, swimming, performance

## Abstract

**Objective:**

This study aimed to investigate the effects of mental fatigue on the perceptual and physiological responses to swimming at the lactate threshold (LT) and on 400-m front-crawl performance.

**Methods:**

Ten national-level swimmers were tested three separate times. In the first session, swimmers performed a 7x200-m incremental test for LT assessment. In sessions two and three, participants performed the AX-Continuous Performance Task for 90-min (mental fatigue condition) or rested for 90-min (control condition) in a randomized and counterbalanced order. After the experimental manipulation, the participants performed a 12x100-m constant-speed test at LT followed by a 400-m front-crawl performance test. Fatigue was measured using the Brunel Mood Scale before and after the experimental manipulation. Heart rate (HR), blood lactate concentration (La) and rating of perceived exertion (RPE) were measured during the swimming tests. Generalized Mixed Models were used to test main effects and interactions, and Holm-Bonferroni post-hoc correction was applied when necessary (*p* < 0.05).

**Results:**

Fatigue increased only for the mental fatigue condition (*p* = 0.018). During the 12 x 100 m constant-speed test at LT, athletes in the mental fatigue condition presented higher RPE (*p* = 0.001) despite similar HR and La responses compared to control. Performance in the 400-m front-crawl test was significantly impaired in mentally fatigued swimmers (*p* < 0.001).

**Conclusion:**

These findings show that mental fatigue increases the perception of effort during swimming at LT despite no significant physiological alterations and reduces 400-m front-crawl performance in national level swimmers.

## Introduction

Traditionally, the study of fatigue in swimming and other sports has focused primarily on the cardiorespiratory, central and peripheral mechanisms of muscle fatigue defined as the exercise-induced reduction in maximal voluntary force or power output of the locomotor muscles (Amann and Dempsey, 2008; Buoite Stella et al., 2024; Yoma et al., 2020). However, coaches and athletes often complain about the negative effects of another kind of fatigue, commonly called mental fatigue (Russell et al., 2019). This is not surprising if we consider that sport is one of the most cognitively and emotionally demanding activities performed by humans (Walsh, 2014). Furthermore, athletes are exposed to various additional psychological stressors related to the sport organization and their personal lives (Mellalieu et al., 2021).

Mental fatigue has been defined as the psychobiological state induced by sustained cognitive effort (Marcora et al., 1985) and it is revealed by subjective feelings of tiredness and lack of energy, brain alterations and impaired cognitive function (Cutsem and Marcora, 2021). Despite plenty of anecdotal evidence from coaches and athletes about the negative effects of mental fatigue on sport performance, only recently researchers has started to investigate this phenomenon (Marcora et al., 1985). The experimental evidence accumulated over the past 15 years suggests that mental fatigue negatively affects endurance performance, cognitive function, technical and tactical performance in physically active subjects and athletes (Cutsem and Marcora, 2021; Habay et al., 2021).

With specific reference to swimming, a study with young swimmers observed a reduction in 1500-m freestyle performance following a 30-min Stroop task in 12 of 16 athletes (Penna et al., 2018). According to the pacing analysis, the participants were slower in the mental fatigue condition in each 300-m split (i.e., 300, 600, 900, 1,200, and 1,500-m). In another study, international-level athletes (FINA score = 616 ± 28) used social media for 30 min to induce mental fatigue, and the effects on the 50, 100, and 200-m freestyle performance were observed (Fortes et al., 2020). For the 50-m race, mental fatigue presented no effect, which is expected for a race that lasts less than 30 s. On the other hand, the races of 100 and 200-m presented a negative effect of mental fatigue. However, the negative effect of mental fatigue on 200-m freestyle performance was not replicated by Quagliarotti et al. (2023) in a group of triathletes. Therefore, more research is needed to confirm the negative effects of mental fatigue on swimming endurance performance. Furthermore, the effects of mental fatigue on the cardiorespiratory, metabolic, biomechanical and perceptual determinants of endurance performance in swimmers are poorly understood.

To fill this gap in the research literature, the first aim of the present study was to investigate the effects of mental fatigue on the heart rate (HR), blood lactate concentration (La) and rating of perceived exertion (RPE) responses to swimming at the lactate threshold (LT). The second aim of the present study was to investigate further the effect of mental fatigue on endurance performance, which was measured with a 400-meter front-crawl test on a group of national-level swimmers. Based on previous findings in other sports (Marcora et al., 1985), we hypothesized (i) a significant increase in perception of effort despite no major cardiovascular and metabolic alterations during swimming at a constant speed, and (ii) a significant reduction in 400-m front-crawl performance in mentally fatigued swimmers.

## Materials and methods

### Participants

As inclusion criteria, participants had to be adult athletes with experience in national or international championships, free from neuromuscular and skeletal muscle injuries or disorders and clear from drugs or medications that could affect physical performance. The participants were excluded if they missed the experimental sessions or refused to follow the study recommendations. Before taking part in the study, all subjects were required to give their written informed consent once the experimental procedures, associated risks, and potential benefits of participation had been explained. The study procedures were approved by the University of Bologna’s Bioethics Committee (protocol: 0126835 from 07/05/2024).

We estimated that 10 participants produced a power of CI_95%_ = 0.990 (0.749–1.00) for the Generalized Mixed Model test. We calculated the power of the study using the app GLIMMPSE^1^. The calculus was made as follows: (a) Hotelling Lawley Trace test; (b) type one error rate of 0.05; (c) 400-m performance as the primary outcome; (d) repeated measures for condition (i.e., MF x CON); (e) no clustering was added to the analysis; (f) no fixed predictors were added; (g) no Gaussian covariate was added; (h) hypothesis contrast of all mean differences zero; (i) theta of zero; (j) smallest group size of 10; (k) marginal means of the CON (i.e., 277-s) and MF (i.e., 288-s) conditions; (l) scale factor for the marginal of 1; (m) standard deviation of 3.2; (n) repeated measure standard deviation ratios of 1 and 2; (o) repeated measure correlation with an unstructured matrix; and (p) scale factor variance of 1.

### Study design

**Figure 1:**
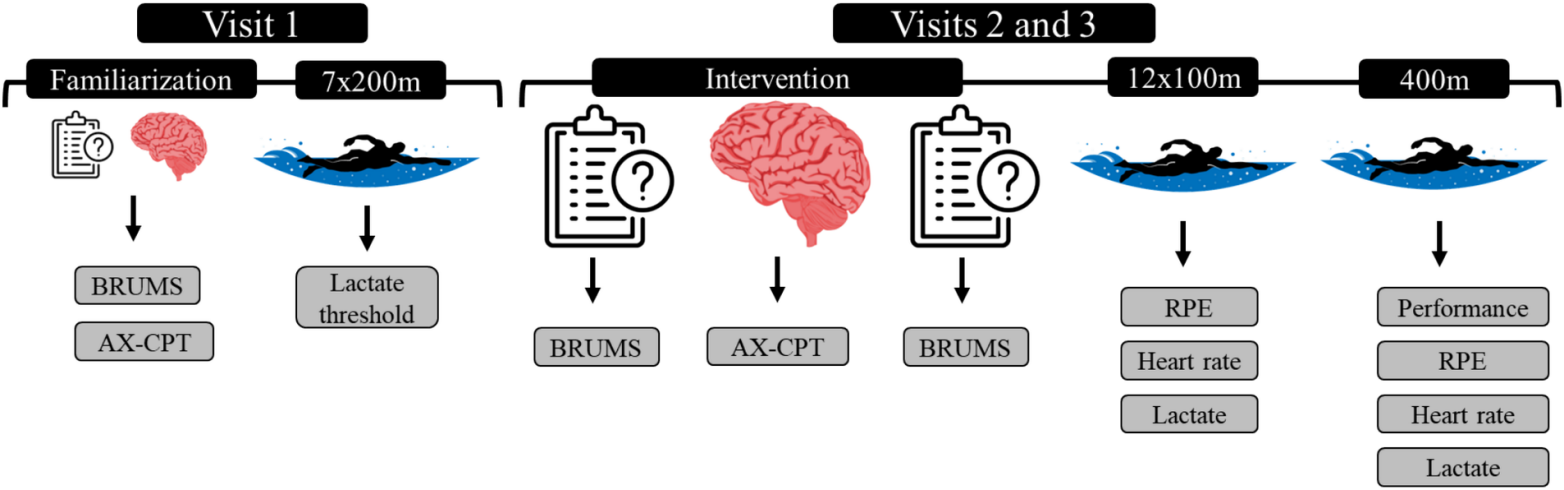
Experimental design of the study. BRUMS = Brunel mood scale; AX-CPT = AX-continuous performance test; RPE = rating of perceived exertion.

The experiment was a single-blind, randomized, counterbalanced AB/BA crossover study with A being the mental fatigue condition and B the control condition. In addition, time (pre and post experimental manipulation) or distance (400, 800, and 1,200-m during the 12 × 100-m constant-speed test) were included as within-subject factors when relevant. Participants were tested three times at the pool where they regularly trained with at least 7 days intervals between testing sessions. During the first session participants were familiarized with all study procedures and performed the 7 × 200-m incremental test to assess LT (see *Swimming tests* section for more details). In the following two sessions, participants performed either the AX-Continuous Performance Task for 90-min (mental fatigue condition) or rested for 90-min (control condition) in the allocated order. Mood was measured before and after the experimental manipulation (see *Experimental manipulation* section for more details). Subsequently, the participants performed the 12 × 100-m constant-speed test followed by the 400-m front-crawl performance test (Penna et al., 2018). During the swimming tests, HR, La and RPE were measured (see *Swimming tests* section for more details). The sessions were planned and organized according to the head coach and their seasonal plan mesocycle to ensure that swimmers received the same training and avoided strenuous exercise sessions 48 h before experimental procedures. All the sessions took place between January and February. An overview of the study design is shown in Figure 1.

### Experimental manipulation

#### Randomization

The participants were randomly assigned to two conditions: mental fatigue and control in a random and counterbalanced order. They were randomized using a random number table with a 1:1 allocation ratio. The allocation was concealed from the researcher who enrolled and assessed participants, and only after the baseline tests, the allocation took place. The researcher who performed the post-intervention measurements was unaware of the condition the participant was allocated to (single blind).

#### Mental fatigue condition

The AX-CPT was used to induce mental fatigue. The task was performed in a silent and bright room, with the participants comfortably sitting on a chair in front of a monitor and wearing an earphone auditive damper to avoid distractions. In this task (Marcora et al., 1985), a sequence of letters, one at a time, was presented to the participants on a computer screen. In front of a response box, participants were instructed to use the mouse and press the right button on target trials and the left button otherwise. The letter “A” appeared as the cue and the “X” as the probe, and the remaining letters served as invalid cues and nontarget probes. The sequences were pseudorandom, with the target (AX) occurring in 70% and nontarget 30% of the time. The letters were centrally presented on a black background for 300 ms in a 24-point Helvetica font, and a 1,200-ms interval followed each letter. A bleep warned the participants when they missed or gave an incorrect response.

#### Control condition

The participants remained in a room for 90-min without any cognitive stimuli. A researcher remained in the experimental room during the task to ensure the subject’s followed the protocol.

#### Manipulation check

Participants responded to the Brunel Mood Scale (BRUMS) (Terry et al., 2003) to assess mood before and after the interventions. The BRUMS consists of 24 items divided into six subscales: anger, fatigue, depression, confusion, vigor, and tension. A Likert scale (5-point) provides a score that ranges from zero to sixteen for each subscale (four items per sub-scale). Based on the mental fatigue phenomenon, only fatigue and vigor subscales were analyzed for the study, as previously used in another study (Marcora et al., 1985).

### Swimming tests

#### 7 × 200-m incremental test

The 7 × 200-m test consisted of seven even-paced swims graded from easy to maximal and performed on a 5-min cycle (Pyne, 2001). Target times were calculated before testing based on 200-m personal best times used as the seventh step’s target time. The target time for the first step was 30-s slower than the target time for the seventh step, with each intermediate step being completed at a speed that is a 5-s faster than the previous step. Each 100-m split and the total 200-m time were timed manually and had to be within 2 s of each other. Blood samples of 5 μL were taken from the earlobe for La analysis (Lactate Pro LT-1710, Arkray, Shiga, Japan) within a 1-min of completion of the swim for the first six efforts and 3 min after the final maximal effort. The anaerobic threshold (AT) was calculated using the D-max procedure (Bishop et al., 1998).

#### 12 × 100-m constant-speed test

A standard warm-up was conducted before the 12 × 100 m. The warm-up consisted of a 300-m front crawl, a 100-m kick front crawl with a kickboard, a 100-m pull with a pull buoy, and a 100-m swim on the front crawl.

Subsequently participants completed one set of twelve 100-m front crawl swims at the speed corresponding to the lactate threshold, with 30 s of rest between repetitions. The hundreds of meters repetitions were performed every fixed time (turnaround), which was the sum of the pace to maintain during their swimming plus the rest (e.g., if the swimmer swam in 1-min and 10-s, with the 30-s rest, the turnaround was 1-min 40-s). Before the start of the test and at the end of the fourth, eighth, and twelfth 100-m repetition, HR was recorded (V800, Polar, Kempele, Finland) and participants were asked to rate their perception of effort using a large 6–20 Borg RPE scale following standard instructions (Pageaux and Lepers, 2016). At the same time, a blood sample of 5 μL was taken from the earlobe for La analysis.

### Statistical analysis

The Generalized Mixed Models (GLzMM) analyzed the main effects and interaction between condition (i.e., control × mental fatigue), time (i.e., pre and post), and distance (i.e., pre, 400-m, 800-m, 1,200-m) for the manipulation check (BRUMS), 12 × 100 test, and 400-m performance. The GLzMM set up as follows: (a) subjects, condition, time, distance, and interactions were tested in the model as random effects; (b) condition, time, and distance as the within-subject variables; (c) Gamma or Gaussian distributions with identity link function for model type; (d) condition, time, and distance as factors; (e) Akaike Information Criterion for the better-fit model; (f) Wald chi-square statistics as the model effects; (g) Holm post-hoc for pairwise comparisons; and (h) graphic analysis of the residuals (Liang and Zeger, 1986). The data are presented in mean and 95% confidence interval of the mean. In case of significant results, the 95% confidence interval of the mean differences (CI95%diff) is presented. The analyses were made using JAMOVI v2.5.3.0.

## Results

### Participants flow

Ten swimmers, six men and four women were recruited to participate in the study and completed it (Table 1). The women in the study were absent from their normal cycle (i.e., amenorrhea). The swimmers were ranked in the top ten nationally in their respective events, with six having competed internationally. No participants were excluded throughout the study.

**Table 1:** Characteristics of the participants.

	Men (6)Mean ± SD	Women (4)Mean ± SD
FINA Points	696 ± 47	716 ± 98
Age (years)	20.6 ± 3.1	21.5 ± 3.8
Height (cm)	184.3 ± 6.2	177.7 ± 7.5
Weight (kg)	81.5 ± 9.2	76.7 ± 7.8
BMI (kg.m^−2^)	23.9 ± 1.8	24.2 ± 1.3

FINA = Fédération Internationale de Natation; BMI = body mass index.

### Manipulation check

#### BRUMS-vigor

**Figure 2:**
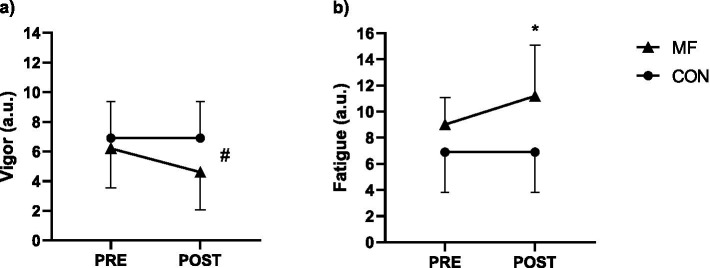
Manipulation check (*n* = 10). Mean and standard deviation of vigor **(a)** and fatigue **(b)**. Generalized Mixed Models, condition (2) × (2) time. * = different from control; # = condition effect.

We observed no condition (*X*^2^_(1,9)_ = 0.4 *p* = 0.526) and interaction effects (*X*^2^_(1,18)_ = 3.5 *p* = 0.061). There was however a significant reduction in vigor over time (*X*^2^_(1,10)_ = 4.7 *p* = 0.029), as seen in Figure 2a.

#### BRUMS-fatigue

We observed no condition (*X*^2^_(1,9)_ = 1.47 *p* = 0.225) effect. Significant results were find for time (*X*^2^_(1,10)_ = 7.11 *p* = 0.008) and interaction (*X*^2^_(1,18)_ = 5.60 *p* = 0.018). We observed increased fatigue following the mental fatigue intervention [PRE = 9.7 (8.3 to 11.1) a.u.; POST = 12.1 (9.7 to 14.6) a.u.; CI_95%diff_ = −2.43 (−4.03 to −0.82); p = 0.018], while in CON fatigue did not change significantly [PRE = 9.0 (5.8 to 12.1) a.u.; POST = 8.6 (5.9 to 11.3) a.u.; CI_95%diff_ = 0.35 (−0.77 to 1.46); *p* = 1.00], as observed in Figure 2b.

### 12×100-m

#### RPE

**Figure 3:**
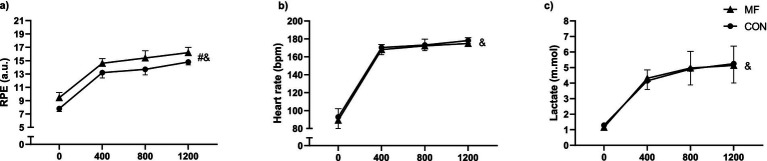
12 × 100-m test. Mean and standard deviation of RPE **(a)**, heart rate **(b)**, and lactate **(c)**. Generalized Mixed Models, condition (2) × (4) distance. RPE = rating of perceived exertion. * = different from control; # = condition effect; & = time effect.

We observed an effect of condition (*X*^2^_(3,63)_ = 54.57 *p* < 0.001) and distance (*X*^2^_(1,63)_ = 786.0 *p* < 0.001), but no interaction effect (*X*^2^_(3,63)_ = 1.65 *p* = 0.645) was found (Figure 3a). MF condition presented increased values for RPE [MF = 13.9 (13.7 to 14.1) a.u.; CON = 12.4 (12.0 to 12.8) a.u.; CI_95%diff_ = 1.49 (1.1 to 1.9); *p* < 0.001].

#### Heart rate

We observed no condition (*X*^2^_(1,16)_ = 2.03 *p* = 0.154) or interaction (*X*^2^_(3,54)_ = 0.353 *p* = 0.950) effects. Only a distance effect (*X*^2^_(1,54)_ = 2608.45 *p* < 0.001) was observed (Figure 3b).

#### Lactate

We observed no condition (*X*^2^_(1,9)_ = 0.001 *p* = 0.974) or interaction (*X*^2^_(3,63)_ = 1.07 *p* = 0.784) effects, only a main effect of distance (*X*^2^_(3,63)_ = 1178.48 *p* < 0.001), as seen in Figure 3c.

### 400-m

#### Performance

**Figure 4:**
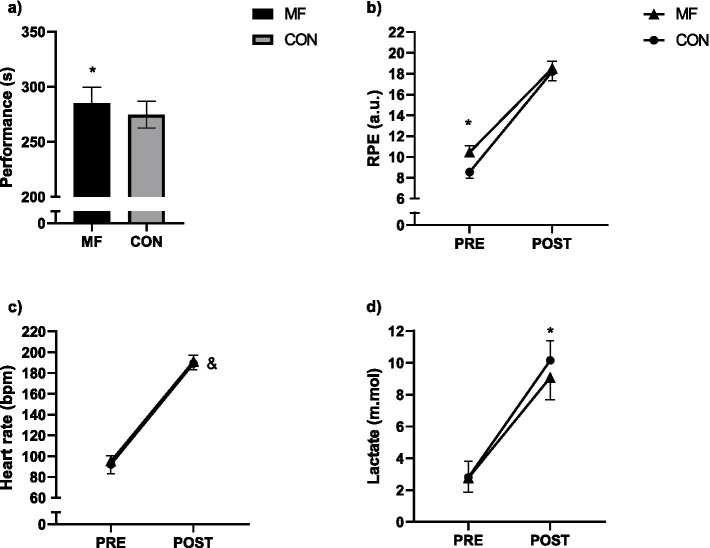
400-m test. Mean and standard deviation of performance **(a)**, RPE **(b)**, heart rate **(c)**, and lactate **(d)**. Generalized Mixed Models, condition (2) for **(a)** and condition (2) × (2) time for **(b,c)**. RPE = rating of perceived exertion; * = different from control; & = time effect.

We observed a condition effect (*X*^2^_(1,9)_ = 117.14 *p* < 0.001). The participants performed better in the CON than in the MF [CON = 277.0 (270.0 to 283.0) s; MF = 288.0 (281.0 to 294.0) s; CI_95%diff_ = −10.89 (−12.86 to −8.92); p < 0.001] condition (Figure 4a).

#### RPE

We observed condition (*X*^2^_(1,27)_ = 8.56 *p* = 0.003), time (*X*^2^_(1,27)_ = 810.2 *p* < 0.001), and interaction effects (*X*^2^_(1,27)_ = 48.29 *p* < 0.001). Participants in the MF condition presented increased RPE values in the pre-measurements [CON = 8.56 (8.12 to 9.00) a.u.; MF = 10.45 (9.97 to 10.93) a.u.; CI_95%diff_ = −9.68 (−10.32 to −9.03); *p* = 0.001]. RPE increased from pre- to post-measurements for the CON [PRE = 8.56 (8.12 to 9.00) a.u.; POST = 18.24 (17.48 to 18.99) a.u.; CI_95%diff_ = −9.68 (−10.32 to −9.03); p = 0.001] and MF [PRE = 10.45 (9.97 to 10.93) a.u.; POST = 18.54 (17.95 to 19.22) a.u.; CI_95%diff_ = −8.08 (−8.74 to −7.42); p = 0.001] conditions. For the post-measurements, the conditions presented similar values [CON = 18.24 (17.48 to 18.99) a.u.; MF = 18.54 (17.95 to 19.22) a.u.; CI_95%diff_ = −0.30 (−1.10 to 0.50); *p* = 0.465] (Figure 4b).

#### Heart rate

We observed no condition (*X*^2^_(1,18)_ = 1.16 *p* = 0.281) or interaction (*X*^2^_(1,18)_ = 0.422 *p* = 0.516) effects, only a time effect was observed (*X*^2^_(1,9)_ = 473.95 *p* = 0.001) (Figure 4c).

#### Lactate

We observed condition (*X*^2^_(1,18)_ = 20.2 *p* = 0.001), time (*X*^2^_(1,9)_ = 70.5 *p* = 0.001), and interaction effects (*X*^2^_(1,18)_ = 36.2 *p* = 0.001). La increased from pre- to post-measurements for the CON [PRE = 3.32 (2.40 to 4.23) mmol/L; POST = 10.24 (9.16 to 11.32) mmol/L; CI_95%diff_ = −7.01 (−8.52 to −5.51); *p* = 0.001] and MF [PRE = 3.15 (2.41 to 3.90) mmol/L; POST = 9.02 (7.93 to 10.10) mmol/L; CI_95%diff_ = −6.92 (−8.42 to −5.41); *p* < 0.001]. Additionally, post-measurements were different between conditions [CON = 10.24 (9.16 to 11.32) mmol/L; MF = 9.02 (7.93 to 10.10) mmol/L; CI_95%diff_ = 1.22 (0.81 to 1.63); *p* < 0.001] (Figure 4d).

## Discussion

We aimed to analyze the effects of mental fatigue on the perceptual and physiological responses to constant-speed swimming and 400-m front-crawl performance. We hypothesize that for the constant-speed test, only the perception of effort will display higher values in the mental fatigue condition with no major cardiovascular and metabolic alterations. A significantly higher RPE during the 12 × 100 m constant-speed test without significant HR and La alterations confirmed this hypothesis. The experiment also confirmed our hypothesis of reduced 400-m front-crawl performance in mentally fatigued swimmers.

In the 400-m test, mentally fatigued participants showed a mean performance reduction of 10 s (3.9%), aligning with findings from similar studies (Fortes et al., 2020; Penna et al., 2018). Penna et al. (2018) observed that following a 30-min Stroop task, young athletes reduced their 1,500-m freestyle performance by 1.2%, whereas in our study, a 3.9% was observed. Our study’s more substantial negative effect might be related to a longer intervention (i.e., 90-min AX-CPT) as the literature suggests that more experienced athletes are less susceptible to the effects of mental fatigue (Martin et al., 2016). Studies should be conducted to clarify this point in the literature. Fortes et al. (2020) also found similar results for the freestyle 200-m, with a difference of approximately 2.0% between conditions. It is important to note that the differences between the first and third place in the 2024 Olympics for the men’s 200, 400, and 1,500-m were 0.04, 0.2, and 0.6%, respectively (FINA, 2024).^1^

For the perceptual and physiological variables in the 400-m, only the lactate presented diverse results compared to literature. The participants in the mental fatigue condition began the test with a higher-than-normal perception of effort, which explains the reduced performance seen in previous studies (de Lima-Junior et al., 2024; Marcora et al., 1985; Pageaux et al., 2015). In mental fatigue studies, the physiological variables usually remain similar in the mental fatigue and control conditions (Marcora et al., 1985; Smith et al., 2016). However, we observed an increase in lactate levels for the control condition. It might be explained by the fact that participants in the control condition could develop more propulsion efficiency during the race due to technical aspects and lower perception of effort. HR (190 bpm), RPE (Russell et al., 2019) and La (11 mmol) at the end of the 400 m test suggest that participants produced a maximal effort during the test. The lower La at exhaustion in the MF condition can be explained by the lower speed produced during the test. The results were as expected in the constant speed 12×100-m test, in which the pacing was controlled during the whole test. Mental fatigue increased perception of effort despite no major cardiovascular and metabolic changes.

Although we consider our findings essential and innovative, some limitations must be mentioned. Our number of participants is small, reducing our generalizability and the possibility of stratifying analyses by sex. Furthermore, a small sample size reduces statistical power for small effects and increases the likelihood of observing large random effects due to higher sampling variability. Therefore, our results should be considered with caution. Nonetheless, our participants are national and international level athletes, which makes the number of ten participants substantial. Also, finding a significant number of high-level athletes who are specialists in a specific swimming technique different from the freestyle is demanding. Our study lacks biomechanical measurements such as stroke rate, stroke length, and propulsive efficiency. This could add interesting information about why 400-m front-crawl performance is reduced in mentally fatigued swimmers.

From a practical point of view, our study suggests that prolonged cognitive effort should be avoided before and between races. A state of mental fatigue should also be avoided before training sessions in which the coach wants to achieve high intensity and volume. However, recent studies suggest that combining physical training with demanding cognitive tasks (Brain Endurance Training, BET) may improve endurance performance (Marcora et al., 2015). Future research should investigate the effects of BET in swimmers. Furthermore, we should investigate the central effects of physical and mental fatigue and its effects on biomechanical and peripheral measurements in order to better understand and avoid performance impairments.

In conclusion, the present data confirms previous findings that mental fatigue impairs the performance of swimmers and suggest that such impairment is caused by an increase in perception of effort rather than significant cardiovascular and metabolic alterations.

## Data Availability

The raw data supporting the conclusions of this article will be made available by the authors, without undue reservation.

## Footnotes

^1^ https://glimmpse.samplesizeshop.org/

^1^ https://www.worldaquatics.com/
